# Corpus Callosum Structural Alterations in Essential Tremor with and Without Resting Tremor: A Multimodal MRI Study

**DOI:** 10.5334/tohm.1083

**Published:** 2025-10-21

**Authors:** Valerio Riccardo Aquila, Maria Celeste Bonacci, Maria Eugenia Caligiuri, Rita Nisticò, Maria Salsone, Andrea Quattrone, Aldo Quattrone, Fabiana Novellino

**Affiliations:** 1 Neuroscience Research Center, University “Magna Graecia”, Catanzaro, Italy; 2 Department of Medical and Surgical Sciences, University “Magna Graecia”, Catanzaro, Italy; 3 Vita-Salute San Raffaele University, 20132 Milan, Italy; 4 IRCCS Policlinico San Donato, San Donato Milanese, 20097 Italy; 5 Institute of Neurology, Department of Medical and Surgical Sciences, Magna Graecia University, Catanzaro, Italy

**Keywords:** Essential Tremor (ET), ET with resting tremor, Corpus Callosum, Thickness, Mean Diffusivity, Fractional Anisotropy

## Abstract

**Background:**

Essential tremor (ET) is a common movement disorder characterized by postural and kinetic tremor. Some patients also show resting tremor, being classified as the ET-plus distinct subtype. The corpus callosum (CC) involvement is proven in several neurological diseases, including ET, but differences between ET with and without resting tremor have not been studied. In this study, we investigated structural characteristics of the CC in a cohort of ET and ET with resting tremor (ETrt) patients, compared to healthy controls (HC).

**Methods:**

We enrolled 128 participants (63 ET, 38 ETrt, and 27 HC). We performed a multimodal MRI evaluation (thickness, mean diffusivity [MD], and fractional anisotropy [FA]) of the CC’s genu, body, and splenium, using different statistical approaches. We first performed a traditional group-based comparison, controlling for relevant covariates. Then, we used an unsupervised classification model based on MRI data to explore potential subgroup distinctions.

**Results:**

Our evaluation showed significant changes in structural parameters of CC in both ET and ETrt patients compared to HC, mainly represented by thickness reductions across all regions and MD increase in the body. Notably, we found no differences between the ET and ETrt groups. Clustering analysis reinforced this observation, placing ET and ETrt in a single cluster with similar abnormalities in all MRI parameters and clearly separating them from HC.

**Discussion:**

Despite their clinical differences, ET with and without resting tremor patients showed analogous macro- and microstructural changes in the CC, suggesting shared pathophysiological processes within this brain region.

**Highlights:**

We explored structural integrity of the Corpus Callosum in ET patients with and without resting tremor. We found a thinning of the corpus callosum and microstructural abnormalities overlapping in ET and ETrt groups, suggesting that despite their different clinical presentations, they share some underlying mechanisms.

## Introduction

Essential Tremor (ET) is the most common neurological cause of tremor. It is primarily characterized by postural and kinetic tremor [1,2], but a variable percentage of patients [3,4] can also manifest resting tremor, a clinical sign traditionally associated with Parkinson’s disease (PD), raising ongoing questions about its significance within the context of ET. It is still a matter of debate if it could represent a sign of a different phenotype of the disease, a late symptom, a sign of a clinical worsening or of a coexisting onset of a PD [4,5]. Anyway, to better classify this clinical variability, the construct of ET-plus has been introduced in the scientific literature to describe patients who present resting tremor or other atypical features alongside the classic manifestations of ET, differentiating them from “pure ET” [4,6], although this has generated considerable debate and has not gained universal acceptance [7]. The neurobiological and pathophysiological mechanisms underlying these two entities remain poorly understood, and there is currently no clear evidence to support a distinct etiological basis. Neuropathological investigations, although primarily focused on the motor cerebellum, have failed to reveal consistent differences between ET and ET-plus, further questioning the validity of ET-plus as a distinct entity [8].

Neuroimaging studies have contributed significantly to our understanding of ET, focusing on the role of the cerebello-thalamocortical network and motor regions, which are thought to underlie tremor pathophysiology. Similarities and differences have been found in ET clinical subtypes, including ET with and without resting tremor, making the physiopathological distinction very difficult to decipher. ET and ET with resting tremor share the involvement of the cerebellar system and networks outside of it, though with some differences. ET with resting tremor appears to have changes that are not limited to cerebellum compared to pure ET, along with additional alterations in deep brain and fronto-temporal regions, suggesting distinct neural features linked to cognitive and motor differences [9,10,11].

Beyond gray matter abnormalities, there is growing evidence of white matter involvement in ET.

Diffusion tensor imaging (DTI) studies have detected microstructural changes in key white matter tracts in ET, and a recent study revealed similar white matter microstructural alterations in ET patients and in ET with resting tremor in cerebellum and several projection pathways, including the basal ganglia and brainstem tracts. However, differences emerged in the degree and lateralization of involvement, particularly within the right and left cerebellar pathways and the dentato-rubro-thalamo-cortical tracts, again highlighting overlap and differences between these disorders [1,12].

Among the white matter bundles, some studies showed the involvement of corpus callosum (CC) in ET [13,14], which is an interesting finding as is the largest commissural fiber bundles responsible for interconnecting the right and left hemispheres [15]. CC acts as a bridge that facilitates communication between regions of opposite lobes that has been found to be altered in neurodegenerative diseases [16,17]. Taken together, neuroimaging findings might suggest that ET with resting tremor could be associated with more widespread changes, involving large-scale brain networks, and a different involvement of right and left networks. CC could play a key role in these bi-hemispherical circuitry. Despite these advances, no study has evaluated any differences in the involvement of the CC, between the ET subtypes.

In this study, we aimed to investigate the structural integrity of the CC in ET and ET with resting tremor (ETrt) patients compared to healthy controls using a multimodal MRI approach. We combined traditional group comparisons with data-driven clustering methods, to identify potential similarities and differences between these subtypes, improving the knowledge on their pathophysiological mechanisms and clinical classification.

## Materials and Methods

### Subjects

We collected data from 128 participants: 63 diagnosed with ET, 38 with ETrt, and 27 HC, all of whom were recruited at the Neuroscience Research Center in Catanzaro, Italy.

All study procedures and ethical aspects were approved by the institutional review board (Magna Graecia University review board, Catanzaro, Italy) and written informed consent was obtained from all the individuals participating in the study.

ET and ETrt were diagnosed according to the established clinical criteria [6]; the clinical diagnosis was confirmed by single photon emission computed tomography (SPECT) with ^123^I-ioflupane, that showed normal uptake in all patients [18]. Moreover, given that the ET-plus category includes a heterogeneous mix of conditions such as dystonia, ataxia, and cognitive deficits, we opted to focus on a more specific subgroup, patients with resting tremor only, to ensure greater clinical consistency within the sample. Therefore, additional inclusion criteria for patients with ETrt were: i) no evidence of dystonia; ii) no evidence of cognitive impairment (MMSE score > 24); iii) no evidence of other neurological signs.

ET and ETrt groups had: a) negative history of neurological or psychiatric disorders other than ET; b) no treatment with deep brain stimulation; c) absence of vascular risk factors; d) no evidence of movement artifacts, vascular lesions, brain tumor, and/or marked cortical and/or subcortical atrophy, and/or diffuse white matter hyperintensities on MRI.

The HC group was selected based on the inclusion criteria of no cognitive decline, as well as no personal or family history of stroke, neurological, or psychiatric disorders.

All participants underwent a careful clinical evaluation, to collect demographic and clinical variables, including age, gender, disease duration and family history. Tremor severity was assessed through Fahn-Tolosa-Marin Scale [19]. For a more accurate assessment of resting tremor, an electromyographic recording of the tremor was performed according to a previously described protocol [20].

In all enrolled subjects, the global cognitive status was assessed by using the Mini-Mental State Examination (MMSE) [21] test; moreover, neuropsychological examination included the Controlled Oral Word Association Test (COWAT), Rey Auditory Verbal Learning Test Immediate and Delayed Recall (RAVLT-IR and RAVLT-DR) and Digit Span forward and backward test [22,23].

### MRI protocol and image analysis

All participants underwent 3T MRI scanning using a GE MR750 scanner with an eight-channel head coil. The imaging protocol included: whole-brain 3D T1-weighted spoiled gradient recalled (SPGR) (BRAVO, TE/TR = 3.7/9.2 ms, flip angle = 12°, voxel size of 1 × 1 × 1 mm^3^) and a diffusion weighted imaging (DWI, voxel size 2 × 2 × 2 mm^3^, matrix size 128 × 128; 80 axial slices; 27 non-collinear directions with gradients at a b-value of 1000 s/mm^2^, 4b = 0 images, NEX = 2). All images were pre-processed using Freesurfer, FSL and an in-house software to analyze the CC profile, as described in previous studies [10,24]. Diffusion-weighted images were processed using FSL’s DTIFit to obtain diffusion tensor imaging (DTI) maps. Image distortions due to eddy currents and head motion were corrected through 3D full affine alignment of each image to the mean no-diffusion weighting (b0) image. DTI data were concatenated into 28 volumes (1 mean b0 + 27 b1000) and the diffusion tensor model was fit at each voxel to generate fractional anisotropy (FA) and mean diffusivity (MD) maps. For each subject, these maps were registered to brain-extracted T1-weighted images using FSL’s FLIRT tools. FA maps were aligned to T1 images using a nonlinear registration with 6 degrees of freedom while other parameters were set using default FLIRT values. The resulting transformation matrix was saved and applied to MD maps to perform alignment to the corresponding T1. Using Freesurfer’s standard recon-all pipeline, we extracted a binary mask of the CC for each subject. Bundle thickness profile was computed following previously established methods [10,24]. In detail, we derived the thickness model by solving Laplace’s equation on callosal voxels [25]. The streamlines from this solution formed non-overlapping, cross-sectional contours, the lights of which were modeled as the callosal thickness. Using a cubic spline interpolation between measurement points, we obtained a smooth centerline that was then divided into 51 parts of equal lengths by 50 nodes. At each node, thickness was measured as the orthogonal distance between CC boundaries. FA and MD values were extracted from 50 regions of interest (ROIs) along the CC centerline by overlaying the thickness profile onto the T1-coregistered DTI maps.

All analyses were conducted on the entire CC, divided into 50 ROIs. To interpret results, we used Witelson’s scheme, which segments the CC into the genu, body, and splenium based on arithmetic fractions of the maximum anterior-posterior extent.

### Statistical analysis

Descriptive statistics were calculated to characterize the sample; we used mean and standard deviation (sd) for normally distributed variables and median and median absolute deviation (mad) for skewed distributions.

The thickness, MD, and FA of the corpus callosum were analyzed in three distinct sections: the genu, body, and splenium. For each section, the average values were calculated for each participant.

We explored possible correlations between MRI measurements and cognitive scores.

To assess differences in means across the groups, we estimated several nested linear regression models, employing Analysis of Variance (ANOVA), the Akaike Information Criterion (AIC), and the Bayesian Information Criterion (BIC) to identify the most suitable model. Our aim was to measure the differences between groups controlling for possible confounding effects, if there were one, derived from age or gender and to quantify the proportion of explained variance by these covariates. The regression models included a null model with only the intercept, a model that included diagnosis as the sole predictor, and more complex models that incorporated age and gender, as well as interactions between these predictors. This stepwise forward selection allowed us to understand if adding another predictor could explain a significant portion of variance.

To ensure that differences in corpus callosum between ET and HC were not a reflection of a global deterioration we extracted white, grey and total brain volumes. We added white matter volumes in all models to account for its effect. Due to multicollinearity, we couldn’t keep all the three volumes as covariates, so we decided to predict thickness, MD and FA of corpus callosum, a predominant white matter structure, with the corresponding white volumes. Furthermore, we tested if there were differences in white, grey and total brain volumes separately with an Analysis of Variance.

Since our primary interest was in differentiating the three participant groups we focused on extracting p-values specifically from the Group variable. For post-hoc analyses, comparisons were conducted between groups for each section of the corpus callosum, with p-values adjusted using the Bonferroni method to control for Type I error across multiple comparisons. Results were presented for each region and MRI measure and differences in the corpus callosum were visualized through the use of boxplots. Furthermore, we verified the assumptions of our models through graphical diagnostics, ensuring the robustness and validity of our findings.

A clustering analysis using the k-means algorithm was subsequently conducted on the MRI data to assess whether an unsupervised learning approach could support our findings. Descriptive statistics were then compared across the identified clusters.

All analyses were conducted with R version 4.3.0. P-values below an alpha level of .05 were considered statistically significant.

## Results

### Participants

Overall, no significant difference was found in patients characteristics (Table 1), nor significant correlations between MRI measurement and cognitive scores. HC, ET and ETrt showed no significant difference in age and gender distribution. The disease duration was slightly shorter in ET compared to ETrt, although this difference was not significant; in the same way, despite differences in the tremor severity measured by Fahn-Tolosa scale were not significant, patients in ET group had lower scores compared to those in ETrt, reflecting the additional burden of resting tremor in the latter group. Regarding cognitive tests, all neuropsychological tests (COWAT, RAVLT and Digit Span) showed the same gradient with HC gaining the highest scores, followed by ET and then ETrt.

**Table 1:** Participants descriptives of demographics, clinical and neuropsychological variables.


	DIAGNOSIS			*p-VALUE*

	HC	ET	ETrt	

**Age**	64 ± 4.5^a^	67 ± 5.9^a^	68.5 ± 12.6^a^	.65^1^

**Gender**	15 M – 12 F	34 M – 29 F	17 M – 21 F	.60*^2^*

**Disease Duration**	/	5.0 ± 5.2^a^	8.0 ± 7.4^a^	.13*^3^*

**Education**	8.0 ± 4.5^a^	9.5 ± 5.2^a^	8.0 ± 4.5^a^	.21*^1^*

**Fahn Tolosa Marin-A**	/	8.0 ± 2.9^a^	12.0 ± 5.9^a^	.68*^3^*

**Postural items**	/	4.0 ± 1.48^a^	4.0 ± 2.22^a^	.98*^3^*

**Kinetic items**	/	3.0 ± 1.48^a^	3.0 ± 1.48^a^	.71*^3^*

**Rest items**	/	/	4.0 ± 1.48^a^	/

**MMSE**	27.7 ± 1.9^b^	27.6 ± 1.6^b^	27.0 ± 1.3^b^	.15*^4^*

**COWAT**	29.7 ± 8.9^b^	26.2 ± 4.6^b^	24.9 ± 8.2^b^	.15*^4^*

**RAVLT_IR**	41.6 ± 5.5^b^	39.5 ± 8.7^b^	36.4 ± 11.1^b^	.74*^4^*

**RAVLT_DR**	8.1 ± 2.1^b^	7.2 ± 2.6^b^	5.9 ± 3.4^b^	.60*^4^*

**Digit Span F**	5.8 ± 1.1^a^	5.2 ± 1.2^a^	4.9 ± 0.9^a^	.09*^1^*

**Digit Span B**	4.0 ± 0.6^a^	3.0 ± 0.7^a^	3.0 ± 0.7^a^	.22*^1^*


*^a^ values are reported as median ± mad; ^b^mean ± sd; ^1^Kruskal-Wallis rank sum test; ^2^Pearson’s Chi-squared test; ^3^ Wilcoxon rank sum test;^4^ANalysis Of VAriance*.

Moreover, no statistically significant differences were found between the three groups in white matter (F = 0.93; p = .398), grey matter (F = 0.29; p = .746) and total brain volumes ( F = 0.59; p = .553).

The assumptions of normality and homoskedasticity of residuals were assessed graphically and were found to be satisfied in all models. Histograms and Q-Q plots indicated that normality of residuals was satisfied. Boxplots of residuals showed a constant variance across the range of predicted values in all three groups. This supports the validity of the statistical analyses conducted and reinforces the robustness of our findings.

### Thickness

**Figure 1:**
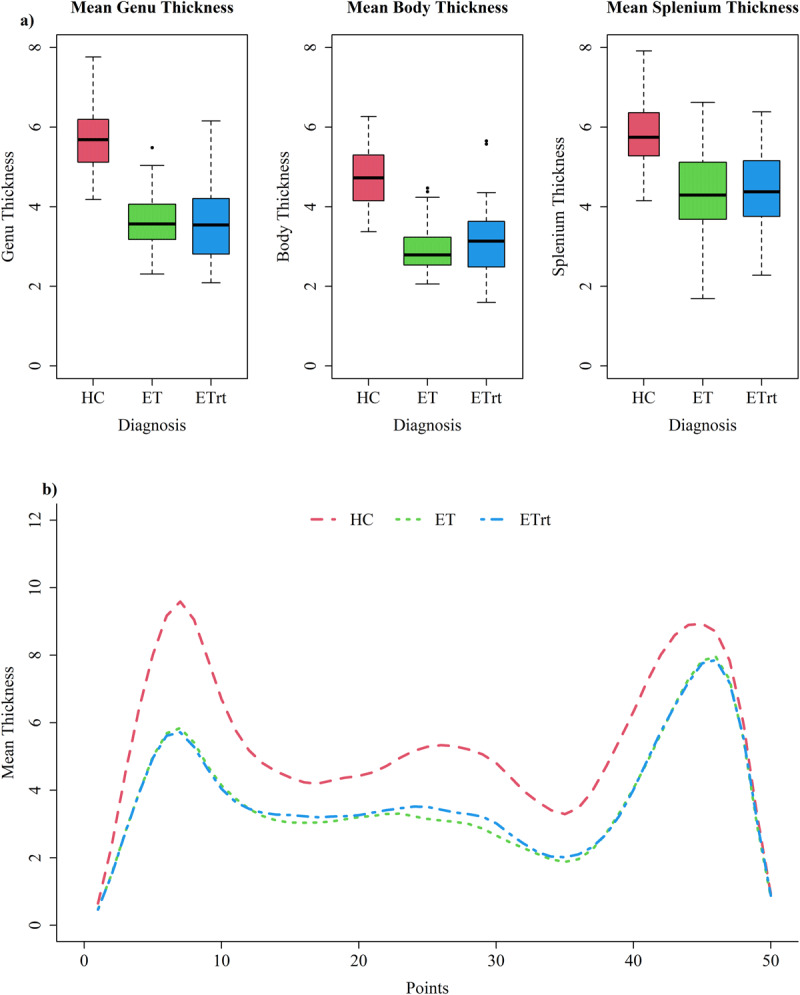
**Mean thickness values in different regions of corpus callosum**. **a)** Boxplots representing mean thickness in different regions of corpus callosum in all groups; **b)** Comparison of mean thickness along all the 50 CC ROIs between the three groups.

Significant differences were observed in thickness between HC and ET patients, as well as between HC and ETrt patients, across all three regions we analyzed (Figure 1). No significant differences were found between the ET and ETrt groups (Table 2). When comparing models in forward selection, age was always considered an important covariate, predicting a decrease in thickness as age increases. Additionally, in the splenium region, it was appropriate to include gender as a covariate (Table 2), with women showing a significantly greater average thickness compared to men. In all the three regions an increase in white matter volumes was associated with higher predicted values in thickness. We decided to keep white matter volumes in all regression models, even when the Bayesian Information Criterion suggested excluding it in some cases. This was necessary to ensure that the predicted differences between groups represented a comparison given equivalent white matter volume. All models in which we included interactions, such as interaction between age and diagnosis or gender and diagnosis, were excluded based on the AIC, BIC, and ANOVA criteria.

**Table 2:** Comparison of Thickness, MD and FA descriptives between the three groups.


	DIAGNOSIS			p-VALUE

	HC	ET	ETrt	

**Genu Thickness (*mm*)^a^**	5.73 ± 0.81	3.66 ± 0.67	3.66 ± 1.01	***HC vs ET p < .001*** ***HC vs ETrt p < .001*** *ET vs ETrt p = 1*

**Body Thickness (*mm*)** ^a^	4.72 ± 0.81	2.95 ± 0.57	3.16 ± 0.90	***HC vs ET p < .001*** ***HC vs ETrt p < .001*** *ET vs ETrt p = 1*

**Splenium Thickness (*mm*)** *^b^*	5.83 ± 0.89	4.40 ± 1.04	4.42 ± 0.89	***HC vs ET p < .001*** ***HC vs ETrt p < .001*** *ET vs ETrt p = 1*

**Genu MD (*mm*** ^2^ ***/s*)** *^b^*	1.7 · 10^–3^ ± 2.3 · 10^–4^	1.6 · 10^–3^ ± 2.5 · 10^–4^	1.7 · 10^–3^ ± 2.9 · 10^–4^	*HC vs ET p = .129* *HC vs ETrt p = 1* *ET vs ETrt p = 1*

**Body MD (*mm*** ^2^ ***/s*)** *^b^*	1.3 · 10^–3^ ± 1.3 · 10^–4^	1.5 · 10^–3^ ± 1.6 · 10^–4^	1.4 · 10^–3^ ± 2.4 · 10^–4^	***HC vs ET p = .029*** *HC vs ETrt p = .07* *ET vs ETrt p = 1*

**Splenium MD (*mm*** ^2^ ***/s*)** ^a^	1.3 · 10^–3^ ± 1.2 · 10^–4^	1.3 · 10^–3^ ± 1.6 · 10^–4^	1.3 · 10^–3^ ± 2 · 10^–4^	*HC vs ET p = 1* *HC vs ETrt p = 1* *ET vs ETrt p = 1*

**Genu FA** ^a^	0.35 ± 0.05	0.39 ± 0.06	0.38 ± 0.07	***HC vs ET p = .033*** *HC vs ETrt p = 1* *ET vs ETrt p = 1*

**Body FA** ^a^	0.43 ± 0.05	0.43 ± 0.05	0.44 ± 0.07	*HC vs ET p = 1* *HC vs ETrt p = 1* *ET vs ETrt p = 1*

**Splenium FA** ^a^	0.55 ± 0.05	0.55 ± 0.05	0.54 ± 0.06	*HC vs ET p = 1* *HC vs ETrt p = 1* *ET vs ETrt p = 1*


*^a^values are reported as mean ± sd; a regression model included Age and white matter volume as necessary covariates;* *^b^regression model included Age and Gender and white matter volumes as necessary covariates*.

### Mean Diffusivity

**Figure 2:**
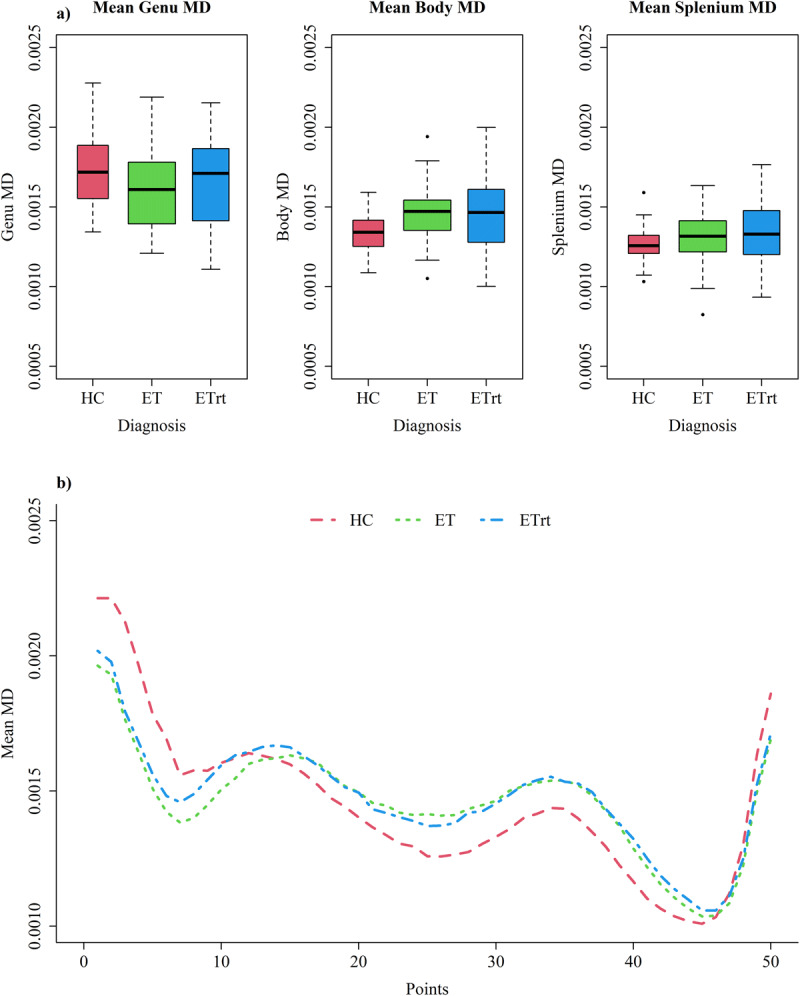
**Mean MD in different regions of corpus callosum**. **a)** Boxplots representing mean MD in different regions of corpus callosum; **b)** Comparison of mean MD along all the 50 CC ROIs between the three groups.

ET patients exhibited higher MD values in the body of corpus callosum compared to HC (Figure 2). It is noteworthy, however, that no significant differences were observed between ET and ETrt groups (Table 2). During the model comparison phase, age and gender were identified as necessary covariates into the genu and body models. Both predictors were associated with an increase in MD; specifically, it was noted that MD scores tend to increase with age, and male participants demonstrated higher MD scores compared to female participants. In the splenium region of the CC, it was necessary to consider only age as a covariate. In all models we had to keep also white matter volumes, specifically higher volumes were associated with lower MD predicted values. Furthermore, all models with an interaction term were excluded based on AIC, BIC and ANOVA criteria. Again, in this case simpler models were preferred to provide an adequate representation of the data without the need for additional complexity and to protect from overfitting. These findings suggest that age and gender could explain a significant proportion of variance in mean diffusivity.

### Fractional Anisotropy

**Figure 3:**
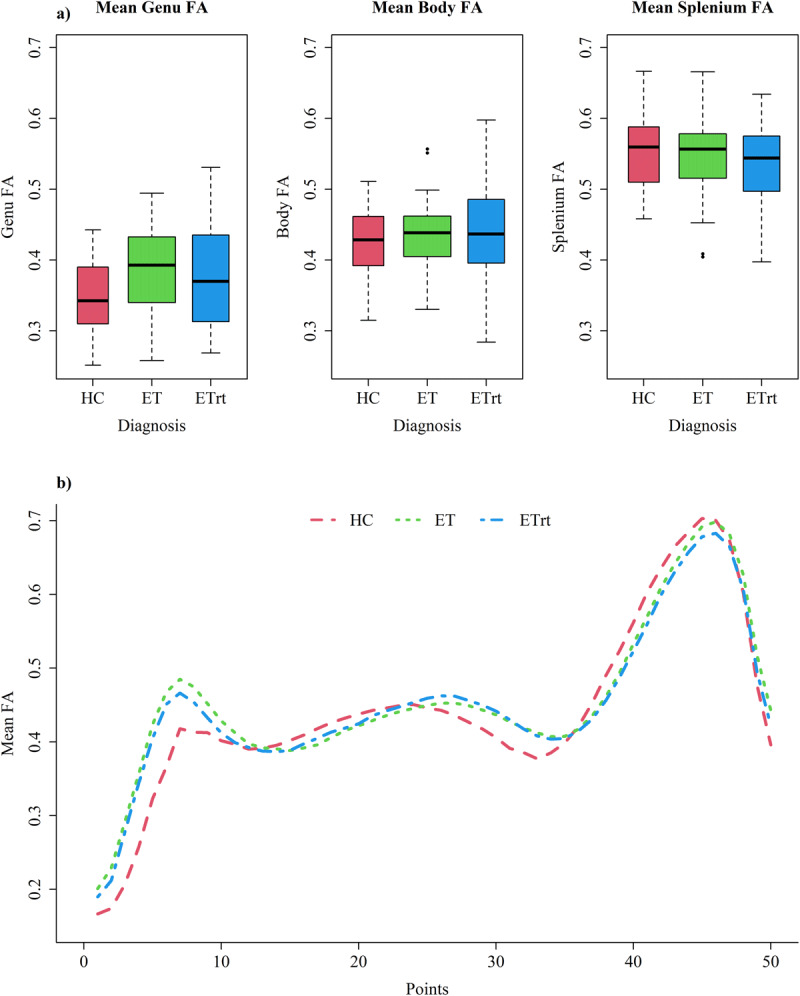
**Mean FA values in different regions of corpus callosum**. **a)** Boxplots representing mean FA in different regions of corpus callosum; **b)** Comparison of mean FA along all the 50 CC ROIs between the three groups.

A statistically significant difference was found in genu FA between HC and ET patients (Table 2; Figure 3). During the model comparison phase, it was determined that age and white matter volumes were necessary covariates for the analysis. Age demonstrated a significant negative relation with FA, while white matter volumes a positive one. The effects of age and white matter volumes, as indicated by the partial eta squared (η*^2^*), were even greater than that of the diagnosis variable (Table 3).

**Table 3:** Effect sizes for the best model in regression analyses.


*MRI PARAMETER REGION*	*PARTIAL ETA SQUARED (η^2^) THICKNESS, MD AND FA*			

	*DIAGNOSIS*	*AGE*	*GENDER*	*WHITE MATTER VOLUMES*

*Thickness*				

*Genu*	*0.53 (0.43, 1)*	*0.09 (0.03, 1)*	*/*	*0.05 (0.01, 1)*

*Body*	*0.48 (0.37, 1)*	*0.09 (0.03, 1)*	*/*	*0.04 (0.01, 1)*

*Splenium*	*0.31 (0.19, 1)*	*0.09 (0.02, 1)*	*0.07 (0.01,1)*	*0.03 (0.00, 1)*

*MD*				

*Genu*	*0.06 (0.01,1)*	*0.30 (0.20, 1)*	*0.06 (0.01, 1)*	*0.09 (0.03, 1)*

*Body*	*0.10 (0.02, 1)*	*0.25 (0.14, 1)*	*0.09 (0.03, 1)*	*0.10 (0.03, 1)*

*Splenium*	*0.03 (0, 1)*	*0.24 (0.14, 1)*	*/*	*0.05 (0.01, 1)*

*FA*				

*Genu*	*0.07 (0.01, 1)*	*0.20 (0.10, 1)*	*/*	*0.13 (0.05, 1)*

*Body*	*0.01 (0, 1)*	*0.12 (0.04, 1)*	*/*	*0.12 (0.04, 1)*

*Splenium*	*0.02 (0, 1)*	*0.18 (0.09, 1)*	*/*	*0.09 (0.03, 1)*


***η^2^ (95% CI)***.

### Clustering Analysis

Since no statistical differences were found between ET and ETrt groups, two clusters were extracted using only the MRI features (thickness, MD and FA) to train the k-means clustering model. The clustering performance was evaluated by assessing whether the data were separated into one cluster primarily composed of ET patients (Cluster 1) and another predominantly consisting of HC (Cluster 2). The results are presented in Table 4.

**Table 4:** Clusters descriptives.


	C1-ET CLUSTER	C2-HC CLUSTER	p-VALUE

**Cluster** **composition**	56 ET 30 ETrt	27 HC 7 ET 8 ETrt	**<.001** ^1^

**Age**	68 ± 6.7^a^	62.5 ± 6.67^a^	**.02** ^2^

**Gender**	39 F 47 M	23 F 19 M	.41^1^

**Genu Thickness**	3.4 ± 0.6^b^	5.4 ± 0.9^b^	**<.001** ^3^

**Body Thickness**	2.8 ± 0.5^b^	4.5 ± 0.8^b^	**<.001** ^3^

**Splenium Thickness**	4.2 ± 0.9^b^	5.8 ± 0.8^b^	**<.001** ^3^

**Genu Mean Diffusivity**	1.7 · 10^–3^ ± 2.4 · 10^–4 b^	1.6 · 10^–3^ ± 3 · 10^–4 b^	.20^3^

**Body Mean Diffusivity**	1.5 · 10^–3^ ± 1.7 · 10^–4 b^	1.3 · 10^–3^ ± 1.7 · 10^–4 b^	**<.001** ^3^

**Splenium Mean Diffusivity**	1.4 · 10^–3^ ± 1.6 · 10^–4 b^	1.2 · 10^–3^ ± 1.5 · 10^–4 b^	**<.001** ^3^

**Genu Fractional Anisotropy**	0.37 ± 0.06^b^	0.38 ± 0.07^b^	.41^3^

**Body Fractional Anisotropy**	0.43 ± 0.05^b^	0.45 ± 0.06^b^	**.03** ^3^

**Splenium Fractional Anisotropy**	0.53 ± 0.05^b^	0.57 ± 0.05^b^	**<.001** ^3^

**Disease Duration**	7.0 ± 5.9^a^	4.0 ± 4.5^a^	.22^2^

**Fahn Tolosa**	9.0 ± 4.5^a^	8.0 ± 4.5^a^	.28^2^

**MMSE**	27.1 ± 1.6^b^	27.9 ± 1.8^b^	**.049** ^3^

**COWAT**	25.6 ± 5.1^b^	29.1 ± 9.7^b^	.12^3^

**RAVLT_IR**	38.9 ± 9.3^b^	41.4 ± 7.1^b^	.21^3^

**RAVLT_DR**	6.9 ± 2.8^b^	8.0 ± 2.6^b^	.10^3^

**Digit Span F**	5.1 ± 0.8^b^	5.6 ± 0.9^b^	**.03** ^3^

**Digit Span B**	3.2 ± 0.8^b^	3.6 ± 0.8^b^	.14^3^


*^a^*values are reported as median ± mad; *^b^* values are reported as mean ± sd; *^1^*Chi-squared test; *^2^*Wilcoxon rank sum test; *^3^*Two sample t-test.

As expected, a significant difference in Diagnosis distribution emerged across the two clusters, with ET/ETrt patients predominantly grouped in one cluster and all HC in the other. For simplicity, we will refer to these as the C1-ET cluster and the C2-HC cluster.

**Figure 4:**
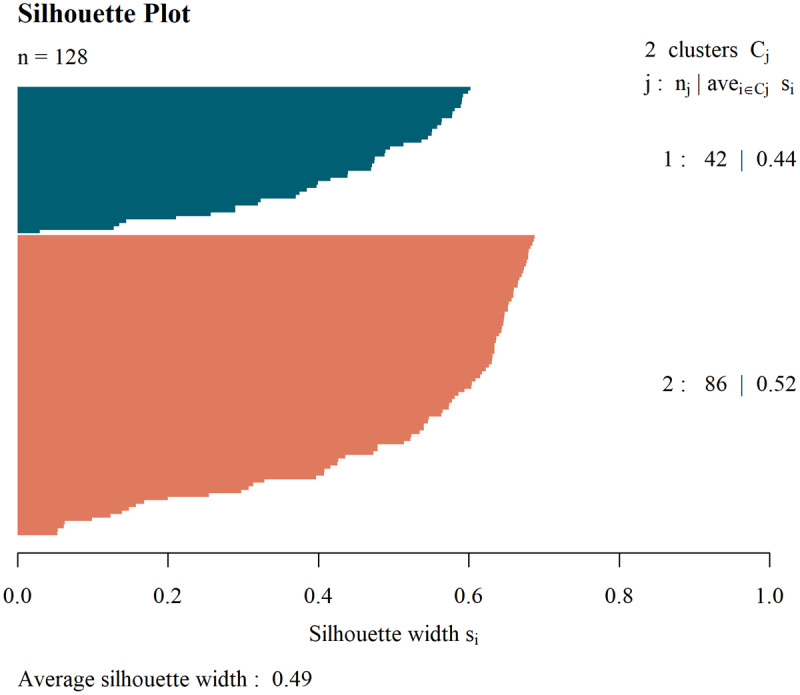
**Silhouette plot of clustering analysis**. The silhouette plot displays the distribution of silhouette coefficients across clusters, indicating the quality of clustering for each data point.

**Figure 5:**
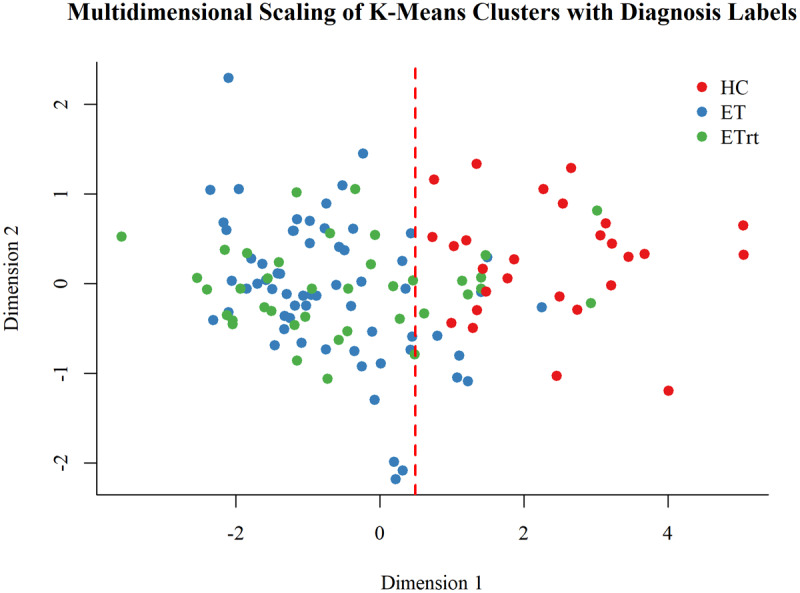
**Multidimensional Scaling representing clusters distributions**. The Multidimensional Scaling represents the spatial distribution of participants based on structural callosal similarity. The dashed line indicates the cluster separation. Each point corresponds to an individual case. The distance between points reflects the degree of dissimilarity. Red dots represent HC, blue dots ET, and green dots ETrt.

Detailed differences between clusters are shown in Table 4. Clusters were different for median age, that was lower in the HC cluster, while we found no significant difference in gender distribution. In line with findings coming from traditional diagnosis-based group comparisons, the clustering-based evaluations showed significant reduction in callosal thickness in ET clusters across all three regions (genu, body, and splenium). ET cluster also showed higher MD and lower FA values in the body and splenium of the corpus callosum, suggesting compromised microstructural integrity. Differences in the genu were not statistically significant for MD nor FA. Among the neuropsychological measures, the two clusters differed for MMSE and Digit Span Forward scores. The effectiveness of the clustering in distinguishing between groups is illustrated in Figures 4 and 5, which provide a graphical representation of the cluster distributions.

## Discussion

This study aimed to evaluate the structural integrity of the corpus callosum in patients with ET and ET-plus with resting tremor compared to HC. Using a multimodal MRI approach, we observed structural changes in all analyzed regions (genu, body, and splenium) in both ET and ETrt patients, without differences between ET groups. Importantly, a data-driven clustering approach based on MRI parameters allowed us to recognize two different callosal profiles, broadly corresponding to HC and ET patients, with ET and ETrt being classified in the same cluster. Overall, converging evidence deriving from our results indicates macro- and microstructural alterations in the corpus callosum of ET patient groups, supporting the notion of an impaired interhemispheric communication, a feature associated with some neurodegenerative diseases. Importantly, ET and ETrt showed an overlapped profile, suggesting that shared neurodegenerative mechanisms affecting white matter integrity underlie both conditions, despite their distinct clinical presentations.

Through a multimodal approach, we were able to detect different callosal abnormalities in patients with ET. Macrostructural evaluations revealed robust and consistent differences in callosal thickness among the three groups. More in detail, all regions of the corpus callosum were significantly thinned out in patients with ET and ETrt in comparison to HC, with the body being the most severely damaged region. In terms of microstructural findings, we found a significant increase in MD of the body of the corpus callosum in both ET patients’ groups. Elevated MD values are considered indicative of reduced global white matter integrity, as in presence of edema, inflammation, or cellular changes that disrupt normal tissue structure. In agreement with our findings, previous studies have reported white matter involvement in ET [26]. In the present study we extended our observations to a cohort of ET patients with resting tremor, demonstrating similar structural abnormalities between the two ET groups. This represents a novel contribution, suggesting that ET and ETrt share a common underlying neurobiological mechanism.

Statistical analysis revealed differences in FA values only in the genu of CC between HC and ETrt, whereas FA values in all other regions did not show alterations among three groups. Our data suggest that the effect of age and WM volume are comparable across cohorts enrolled. The increased FA value observed in the genu of CC in ETrt compared to HC could not reflect pathological alterations, but could be influenced by specific fiber composition. Indeed, fiber in this region are typically less myelinated, thinner, shorter and more widely fanned than those in the rest of the CC. These structural characteristics might enhance the directionality of diffusion, thereby contributing to observed an increase of FA. However, MD and FA provide complementary information about fibers in the brain: MD quantifies the mean diffusion of water, providing an estimation of the density and compactness of white matter; FA evaluates the preferred direction of diffusion, indicating the orientation and integrity of nerve fibers [27]. Therefore, when the damage is subtle, the overall directional movement (anisotropy) can be maintained, leading to normal or near-normal FA values [28,29,30].

The corpus callosum was previously studied in ET subjects with different results.

A study did not reveal significant alterations in the genu of corpus callosum comparing MD and FA between ET and HC [14]. On the contrary, other studies have found decreased FA and increased MD in several white matter bundles, including the corpus callosum, with white matter measures correlating with neuropsychological scores [31,32]. Similarly, another study [13] reported increased MD in the corpus callosum of ET patients, with significant correlations between MD values and neuropsychological test scores. Some abnormalities in MD were found in tracts involved in primary motor functions in ET patients, but they did not find significant correlations between DTI measures and clinical rating scale scores [33]. Moreover, ET patients showed significantly lower values of fiber density, fiber bundle, and fiber density bundle cross-section compared to PD [34].

In the present study, to better capture the neuroanatomical patterns distinguishing HC from ET patients we performed a data-driven clustering analysis using only MRI-derived parameters of corpus callosum. This approach added significant information and helped us to verify the existence of structural differences from another perspective. Indeed, the unsupervised analysis successfully allowed us to clearly distinguish two clusters: a cluster including most patients with ET and ETrt (C1-ET cluster) and a second cluster comprising all the HC (C2-HC cluster). Interestingly, ET and ETrt individuals were mostly grouped together in the same cluster showing significantly reduced callosal thickness, elevated MD, and lower FA in both the body and splenium, further proving a widespread callosal involvement in ET, that is similar in both groups of ET patients. Interestingly, while traditional group-level comparisons failed to detect significant differences in FA values in both the body and splenium, these changes emerged by using clustering approach. Subtle but significant disparities between clusters also emerged in MMSE and Digit Span Forward scores, suggesting that structural changes may be accompanied by mild cognitive alterations. The emergence of these differences between clusters may be due to the ET and ETrt being merged into the same group, forming a larger one, thus increasing the statistical power and making the tests more sensitive to detect small differences.

At the same time, clustering separation wasn’t perfect. A few ET patients, again in the same proportion of both ET and ETrt subjects, were included in the cluster with all the HC subjects. This could reflect a variability within the ET population or possibly milder disease forms.

Our findings add to the ongoing discussion about the structural neural features of resting tremor in the context of the ET-plus phenotype and its differences from pure ET. Some authors suggest that ET-plus may not constitute a clinical entity separate from the ET, and rather, it might represent a spectrum of clinical variability within the disease, potentially reflecting factors such as disease progression or individual symptom heterogeneity [35,36,37].

From a neuroimaging perspective, several studies have explored structural and functional differences between ET patients with and without rest tremor. A number of studies have demonstrated that alterations in basal ganglia circuits appear predominant in patients exhibiting rest tremor [10,38]. However, converging evidence coming from different studies with different approaches suggest that both clinical phenotype share the cerebellar involvement as the central pathophysiological hallmark [9,12,39], thus demonstrating overlapping pathogenic mechanisms underlying both presentations.

In our study, we focused on changes in the corpus callosum and found a similar pattern of involvement between the two tremor subtypes, reinforcing the idea that ET with resting tremor and “pure” ET share common neurobiological substrates and supporting the notion that ET with and without resting tremor could represent distinct subtype of the same clinical entity.

Our results showed no correlations between clinical and neuropsychological evaluations and MRI measures in ET groups, but we found a role of gender and particularly of age, which was a strong covariate across all models. In line with previous observations in the general population [40], as the age increases, the callosal thickness decreased in both patients and healthy controls groups. Moreover, we found that women had enhanced callosal thickness and decreased MD values respect to the men in the splenium. Considered together, this evidence suggests that age and gender may influence the structure of CC and this should be considered when interpreting results from heterogeneous populations.

The main limitation of this study was the sample size. Despite it was overall reasonably large, the relative proportions of ET, ETrt and HC were not balanced. This aspect could potentially influence the generalizability of the findings. However, this study also has several strengths. Firstly, the careful clinical selection of patients, particularly regarding ET diagnosis and the assessment of resting tremor. In addition, to obtain robust results, we used an extremely rigorous statistical approach. Finally, neuroimaging analysis was conducted using strict methodological criteria, adding robustness to our structural findings. Future studies with larger, more balanced samples will confirm these results.

In conclusion, this study aimed to explore structural features of the corpus callosum in ET patients with and without resting tremor, using a multimodal MRI approach and double hypothesis-driven and data-driven methods. A key strength and novelty of our study lies in the combined use of a traditional diagnosis-driven group comparison and an unsupervised, data-driven clustering analysis. This dual statistical approach allowed us not only to test differences based on the diagnostic group, but also to explore intrinsic patterns in the MRI data independently of clinical labels. Results deriving from both approaches demonstrate significant macro- and microstructural alterations in the CC in both ET and ETrt patients compared to HC, with no distinct separation between the two patient groups, suggesting shared neuroanatomical features despite clinical distinctions.
